# Management of chronic subdural hematoma: A study from Jordan

**DOI:** 10.1016/j.jtumed.2022.06.008

**Published:** 2022-07-09

**Authors:** Sultan Jarrar, Mohammed M. Al Barbarawi, Suleiman S. Daoud, Qais A. Samara, Aref A. Qarqash, Rama J. Alawneh, Nancy A. Abu-amoud, Obada E. Ababneh, Omar F. Jbarah

**Affiliations:** a Neuroscience Department, Division of Neurosurgery, Faculty of Medicine, Jordan University of Science & Technology, Irbid, Jordan; b Faculty of Medicine, Jordan University of Science and Technology, Irbid, Jordan

**Keywords:** ورم دموي تحت الجافية مزمن, عوامل التنبؤ, نزح بر-هول, نكسة, الأردن, Burr hole drainage, Chronic subdural hematoma, Jordan, Predictors, Recurrence

## Abstract

**Objectives:**

Chronic subdural hematoma (CSDH) is a common condition encountered in neurosurgical practice. Few studies have reported the characteristics of CSDH patients in the Middle Eastern population. We describe the clinical presentation, surgical management, radiological findings, and post-operative outcomes in our hospital.

**Methods:**

We performed a retrospective cohort study in King Abdullah University Hospital, Northern Jordan, between 2009 and 2019. Data were extracted from patients’ medical records and analyzed in patients treated with burr hole drainage (BHD). Univariate analysis was performed to identify correlations with age, laterality, and recurrence.

**Results:**

A total of 172 CSDH patients were identified, of whom 128 (74.4%) were treated surgically. The mean age of patients treated with BHD (n = 108) was 60.9 years with a male-to-female ratio of 2.38:1. Headache was the most common presenting symptom (64.81%) and was significant in patients aged 41–64 years (p = 0.004), whereas muscle weakness and unsteady gait were significant in patients ≥ 65 years (p = 0.004 and p = 0.033, respectively). A higher pre-operative maximum thickness was associated with bilateral presentation (p = 0.001), whereas a higher pre-operative midline shift was associated with unilateral presentation (p = 0.027). Regarding CSDH recurrence, only a preoperative midline shift was significant (p = 0.021).

**Conclusion:**

Clinical presentation was affected by age, as patients < 65 years commonly presented with headaches, whereas those ≥ 65 years presented with limb weakness, speech impairment, unsteady gait, and altered consciousness. BHD was the most utilized surgical option with low mortality and complication rates. Recurrence was only associated with a pre-operative midline shift.

## الملخص

**أهداف البحث:**

الورم الدموي تحت الجافية المزمن هو حالة شائعة في ممارسة جراحة الأعصاب. ذكرت دراسات قليلة خصائص مرضى الورم الدموي تحت الجافية لدى سكان الشرق الأوسط. تهدف الدراسة إلى وصف العرض السريري، والإدارة الجراحية، والنتائج الإشعاعية، ونتائج ما بعد الجراحة في مستشفانا.

**طرق البحث:**

أجرينا دراسة جماعية بأثر رجعي في مستشفى الملك عبد الله الجامعي، شمال الأردن، في الفترة من 2009 إلى 2019. تم استخراج البيانات من السجلات الطبية للمرضى وتحليلها عند المرضى الذين عولجوا بطريقة "نزح بر-هول". تم إجراء التحليل أحادي المتغير لتحديد العلاقة مع العمر والتجانب والنكسة.

**النتائج:**

تم تحديد 172 مريضا، منهم 128 (74.4٪) عولجوا جراحيا. كان متوسط عمر المرضى الذين عولجوا بطريقة "نزح بر-هول" (عددهم 108) 60.9 سنة مع نسبة ذكر إلى أنثى 2.38: 1. كان الصداع أكثر الأعراض شيوعا (64.81٪). من ناحية أخرى، كان ضعف الأطراف، وضعف الكلام، والمشية غير المستقرة مهمة إحصائيا عند المرضى الأكبر من 65 سنة. كما ارتبطت السماكة القصوى قبل الجراحة بالمظهر السريري ثنائي الجانب، بينما ارتبط تحول خط الوسط قبل الجراحة بالمظهر السريري أحادي الجانب. فيما يتعلق بالنكسة، كان تحول خط الوسط قبل الجراحة فقط ذا دلالة إحصائية مهمة.

**الاستنتاجات:**

تأثر المظهر السريري بالعمر، حيث أن المرضى الذين تقل أعمارهم عن 65 عاما يعانون من الصداع، بينما يعاني الذين تزيد أعمارهم عن 65 من ضعف الأطراف، وضعف الكلام، المشية غير المستقرة، والوعي المتغير. كانت طريقة "نزح بر-هول" هي الخيار الجراحي الأكثر استخداما مع معدل منخفض للوفيات والمضاعفات. فيما يتعلق بالنكسة، كانت مرتبطة فقط بتحول خط الوسط قبل الجراحة.

## Introduction

Chronic subdural hematoma (CSDH) is an encapsulated collection of blood and fluid underneath the dura mater. It is one of the most common neurosurgical conditions with an overall incidence rate ranging from 1.72 to 20.6 per 100,000/year, and an estimated 48/100000 persons per year in patients ≥ 65 years old.^1^^,^^2^ Diagnosis is based on either computed tomography (CT) scan or magnetic resonance imaging (MRI). Conservative management with steroids, atorvastatin, tranexamic acid, and other antifibrinolytic medications is sometimes indicated for asymptomatic patients or small hematomas, whereas surgical intervention is preserved for symptomatic or large hematomas.^3^, ^4^, ^5^, ^6^ Burr hole drainage (BHD), whether single or multiple, is the preferred surgical technique in our institute. Other alternative techniques include craniotomy or twist drill craniostomy.^7^

Recurrence of CSDH is a common complication after surgery with rates ranging from 5% to 30%.^8^, ^9^, ^10^, ^11^ Several risk factors are associated with recurrence, including hematoma density and thickness,^12^^,^^13^ pre- and post-operative midline shift,^12^^,^^14^, ^15^, ^16^ bilateral CSDH,^17^^,^^18^ use of anti-coagulant or anti-thrombotic therapy,^12^^,^^19^^,^^20^ type of surgical procedure,^9^^,^^21^^,^^22^ Glasgow Coma Scale (GCS) score,^10^^,^^23^ and comorbidities (e.g., hypertension [HTN] and diabetes mellitus [DM]).^12^^,^^17^

Few studies have described CSDH in the Middle East.^24^^,^^25^ This study describes the clinical presentation, radiological findings, surgical management, and post-operative outcomes in patients with CSDH over a 10-year period in King Abdullah University Hospital (KAUH), a tertiary hospital in northern Jordan.

## Materials and Methods

### Patients

We collected the medical records of patients diagnosed with CSDH who were treated conservatively or surgically at the Division of Neurosurgery, Department of Neuroscience of KAUH, from January 2009 to December 2019. A diagnosis of CSDH was confirmed by CT scan or MRI. Exclusion criteria included patients who were diagnosed with other intracranial hemorrhages concurrent with CSDH, hygroma cases, and history of CSDH evacuation outside KAUH.

### Data extraction

We extracted information regarding patients’ age, sex, location of CSDH on brain lobes, laterality (right, left, or bilateral), CT density, history of smoking, comorbidities, surgical modalities, pre- and post-operative steroid use, pre- and post-operative GCS, pre- and post-operative midline shift (mm), pre- and post-operative maximum thickness on CT scan (mm), surgical complications, and ongoing medications. In the case of bilateral CSDH, maximum thickness was calculated as the sum of right and left hematomas. Density was classified relative to the brain density into four categories: hypodensity, isodensity, hyperdensity, and mixed density. The definition of recurrence was adopted from Chen et al.^26^ as subsequent radiographic changes following the primary treatment, with re-bleeding and/or increased size of the subdural hematomas on the operated side, with or without any clinical presentation.

### Surgical indications and management

Single or double burr holes with a closed-system drainage were indicated in cases of symptomatic CSDH. In addition, midline shift should be >0.5 cm and/or maximum hematoma thickness of >1 cm to indicate surgical intervention. Patients underwent either local or general anesthesia. A small skin incision (3.8 cm) was made in the scalp straight down to the bone. A hole was drilled through the bone revealing the dura mater. After opening the dura mater, any fluids in the subdural space were drained. Irrigation with normal saline was carried out in all cases. The drain was removed 48–72 h after surgery. All anti-coagulants and anti-platelets were discontinued before the operation, and we ensured that the international normalized ratio (INR) was less than 1.3.

### Statistical analyses

We analyzed patients who were treated surgically. Descriptive measures included counts and proportions for categorical data (%), and means (standard deviations [SDs]) and medians with interquartile ratios (IQRs) for continuous data. The chi-square test or Fisher's exact test if one cell count was less than 5, was used to analyze associations between the categorical variables. The Student's *t*-test and analysis of variance (ANOVA) were used for normally distributed continuous variables, and the Mann–Whitney U test and Kruskal–Wallis test were used for non-normal distributions. Normally distributed continuous variables were examined using the Shapiro–Wilk test. Effect sizes used were Cramer's V for categorical data, r statistics for the Mann–Whitney U test, Cohen's d for Student's *t*-test, and eta-squared (η^2^) for ANOVA and Kruskal–Wallis test, considering (0.10 to <0.30) as a small effect, (0.30 to <0.50) as a medium effect, and (≥0.50) as a large effect for both Cramer's V and r statistics; (0.20 to <0.50) as a small effect, (0.50 to <0.80) as a medium effect, and (≥0.80) as a large effect for Cohen's d; and (0.01 to <0.06) as a small effect, (0.06 to <0.14) as a medium effect, and (≥0.14) as a large effect for eta-squared (η^2^).^27^^,^^28^ The 95% confidence intervals (CIs) of Cramer's V were calculated using the boot-strapping method, setting the number of replicate samples to 1000, and the 95% CIs of Cohen's d and eta-squared (η2) were calculated using the non-central t and f distributions, respectively. The age variable was further divided into three subgroups when conducting the analyses (≤40 years, 41–64 years, and ≥65 years), based on what was previously reported by Won et al.^29^ The pair-wise case deletion method was used for data missing at random. Two-sided P ≤ 0.05 was considered statistically significant. All statistical analyses were done using SPSS software (version 26; IBM Corporation, Armonk, NY, USA).

## Results

### A- General demographics

A total of 172 CSDH cases were identified in our institute, of which 128 (74.4%) were treated surgically and 44 (25.6%) were treated conservatively. The overall mean age was 54.9 years, with a male predominance of 67.4% (116/172). Of the 128 patients treated surgically, 17 underwent craniotomy, 108 underwent BHD, and 3 were excluded due to a history of evacuation outside KAUH. Finally, 108 patients were included in this study.

### B- Burr hole drainage

**Figure 1:**
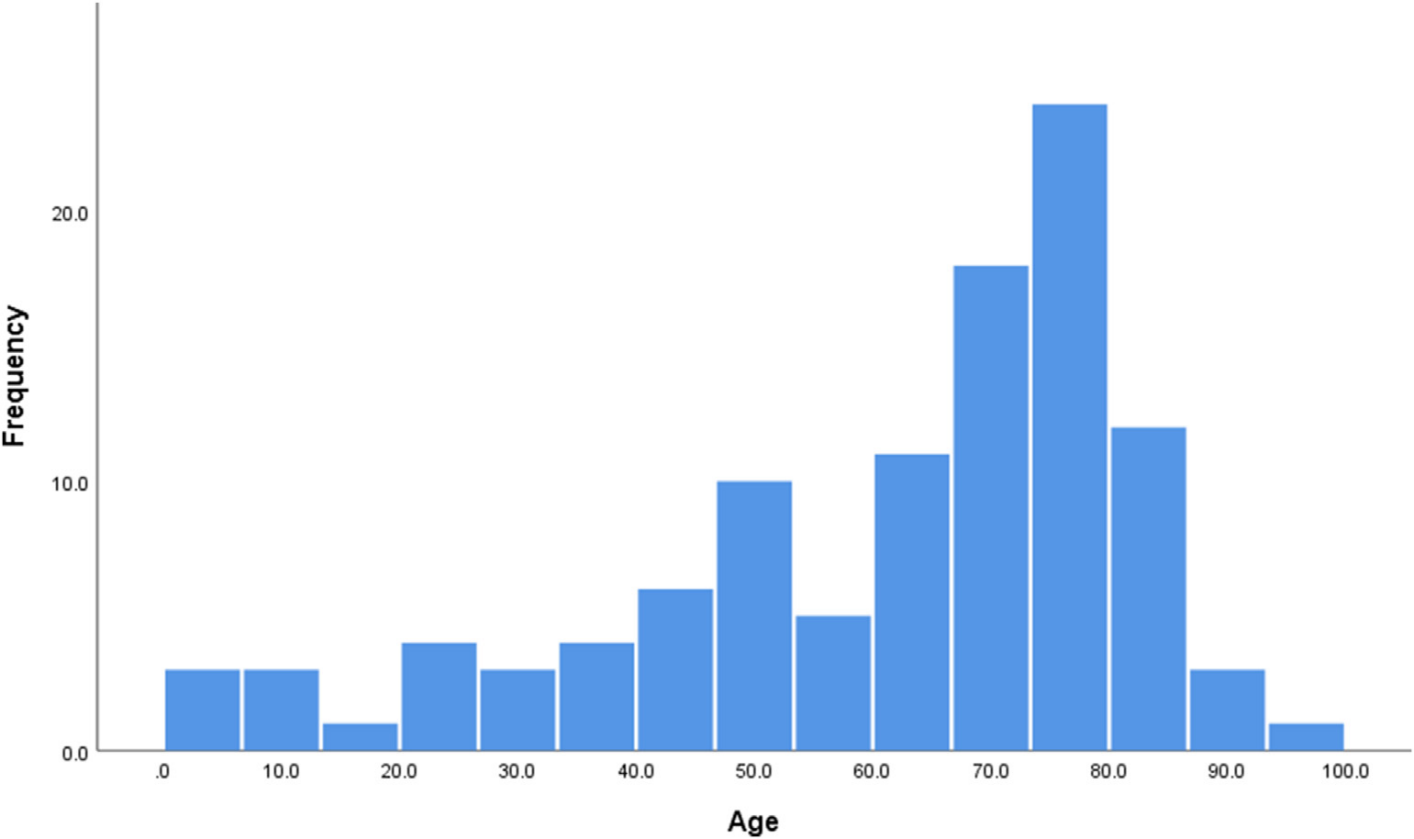
Histogram with the distribution of age groups for patients diagnosed with chronic subdural hematoma and managed with burr hole drainage.

A total of 108 patients who underwent BHD were identified, of whom 88 (81.5%) were treated using double BHD, with a mean age of 60.9 years and a male predominance of 70.4% (76/108). The proportion of patients ≥ 65 years and patients < 18 years were 58.3% (63/108) and 6.5% (7/108), respectively. Of the patients < 18 years, three were less than 1 year old. A histogram with the distribution of ages is presented in Figure 1.

The most common clinical complaints of the 103 patients with available data were headache (64.8%), limb weakness (40.8%), nausea/vomiting (30.1%), and altered consciousness (20.4%). The most common comorbidities of the 107 patients with available data were HTN (55.1%), heart diseases (36.5%), and DM (33.7%). In addition, cerebrovascular diseases and non-vascular neuropathies were found in 24 (22.4%) and 15 (14%) patients, respectively. Regarding the pre-operative history of medications, 35 (32.7%) and 12 (11.2%) patients (n = 107) were on anti-platelet and anti-coagulant drugs, respectively, with only 7 (6.5%) patients taking both. Only eight (13.56%) patients were on HTN medications when presented to the hospital. Traumatic accidents were identified as a direct cause of CSDH in 29 (27.1%) patients.

CT and MRI scans were performed as diagnostic modalities in 97% and 3% of cases, respectively. CT scan densities were mostly mixed density (89%) with no cases of hyperdense hematomas. Regarding laterality, 68.5% of cases were unilateral and 31.5% were bilateral. The most common locations affected in both were the parietal (96% and 92%, respectively) and frontal (92% and 94%, respectively) lobes. Full details of locations affected are available in Table 1.

**Table 1:** All locations affected.

	Unilateral	Bilateral
	74	34
Frontal lobe	68	32
Parietal lobe	71	32
Temporal lobe	27	20
Occipital lobe	33	16
Tentorium	2	7
Central falx	3	7
Posterior fossa	1	0

The pre-operative GCS was mostly mild (13–15) (78.9%). Regarding the maximum thickness and midline shift on CT scans, only 70 and 68 patients, respectively, had available data. The pre-operative maximum thickness (in mm) had an overall mean of 26.2 ± 12.6, whereas the post-operative maximum thickness had an overall mean of 14.5 ± 9.5. Regarding pre-operative midline shift, the overall mean was 7.4 ± 5, whereas the post-operative midline shift had an overall mean of 2.6 ± 2.6.

**Figure 2:**
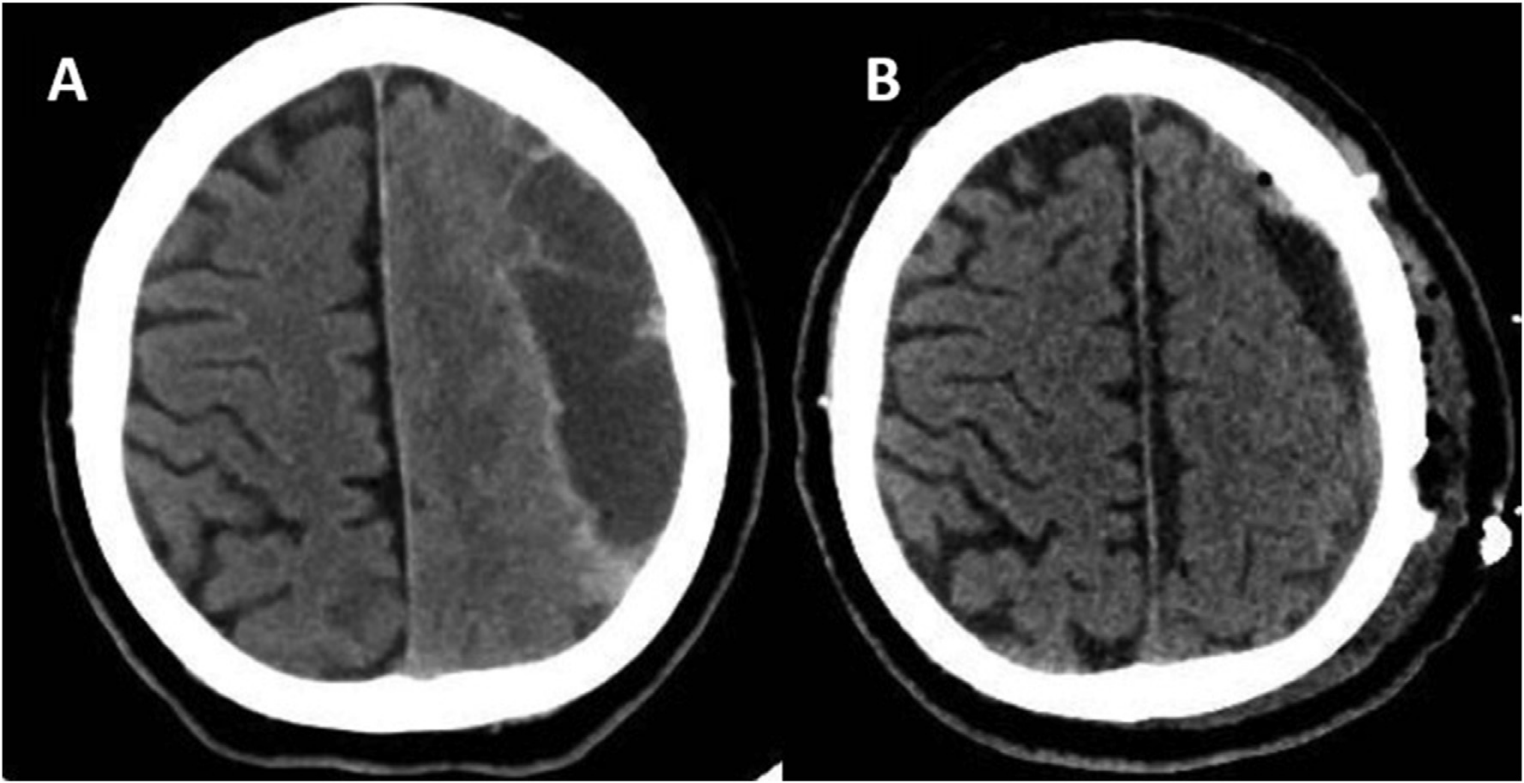
Example of a brain computed tomography scan of a 75-year-old male before and after successful burr hole drainage.

Lastly, recurrence occurred in only 14 of the 108 patients (13%), complications were experienced in 10 (9.3%) patients, and the mortality rate was 2.8% (3/108). Figure 2 shows an example of non-contrast CT scan of a 75-year-old male before and after successful BHD.

#### I. Comparisons based on age

Upon grouping the patients based on age, the mean age was 20.8, 52.6, and 75.9 years in the ≤40 years, 41–64 years, and ≥65 years groups, respectively. Of the patients with maximum thickness data available, the older age group (M = 28.6, SD = 10) had a higher mean pre-operative maximum thickness than both the 41–64 and the ≤40 groups, with the latter having the lowest of the three (M = 24.3 and 20.2, SD = 16.2 and 12.9, respectively) with statistical significance and a medium effect, p = 0.016 (η^2^ = 0.11, 95% CI: 0.00–0.24). Similarly, when comparing the post-operative maximum thickness, the older age group (M = 17.1, SD = 10.1) also had a significantly higher mean than the other two age groups (M = 10.6 and 11.5, SD = 7.3 and 8.2, respectively) and a medium effect as well, p = 0.035 (η^2^ = 0.09, 95% CI: 0.00–0.22).

Regarding the patients with midline shift data, the means of the pre- and post-operative midline shift did not significantly differ.

Regarding the clinical presentation, the 41–64 year group had a significantly higher proportion of patients reporting headache than the other two age groups (55.7% for the older age group vs. 91.7% for the 41–64 group vs. 77.8% for the younger age group; V = 0.33 “medium effect” [95% CI: 0.18–0.49], p = 0.004), whereas the older age group had a significantly higher proportion reporting muscle weakness (54.1% vs. 25.0% vs. 16.7%, respectively; V = 0.33 “medium effect” [95% CI: 0.18–0.51] p = 0.004), unsteady gait (19.7% vs. 4.2% vs. 0.0%, respectively; V = 0.26 “medium effect” [95% CI: 0.15–0.38], p = 0.033), and altered consciousness (29.5% vs. 8.3% vs. 5.6%, respectively; V = 0.27 “medium effect” [95% CI: 0.14–0.42], p = 0.026).

Concerning the comorbidities, HTN, DM, and heart diseases were all statistically significant, with the older age group having the highest proportion (74.2% vs. 48.1% vs. 0.0%, respectively, V = 0.55 “large effect” [95% CI: 0.34–0.68], p ≤ 0.001; 45.2% vs. 29.6% vs. 0.0%, respectively, V = 0.35 “medium effect” [95% CI: 0.26–0.47], p = 0.001; and 46.8% vs. 33.3% vs. 5.6%, respectively, V = 0.31 “medium effect” [95% CI: 0.19–0.46], p = 0.006).

The use of anti-platelets was observed in both the older age group and the 41–64 years group, but not in the younger age group (35.5% vs. 48.1% vs. 0.0%, respectively; V = 0.33 “medium effect” [95% CI: 0.25–0.47], p = 0.003), whereas the use of anti-coagulants did not significantly differ.

All of the results with their effect sizes for comparisons based on age are available in Table 2.

**Table 2:** All age-based comparisons effect sizes.

		Total	≤40	41–64	≥65	p-value	Effect sizes (95% CI)
			N = 18	N = 27	N = 63		
Age	Mean (SD)	60.9 (22.1)	20.8 (13)	52.6 (7.2)	75.9 (6.9)		
	Median (Q1–Q3)	68.5 (48.3–76)	23.5 (10.5–30.5)	51 (47–60)	76 (71–81)		
	Min–Max	0.3–98	0.3–39	42–63	65–98		
Sex	Male	76 (70)	11 (61)	17 (63)	48 (76)	0.290	0.15 (0.04–0.36)
Laterality	Unilateral	74 (69)	11 (61)	19 (70)	44 (70)	0.759	0.07 (0.03–0.31)
	Bilateral	34 (32)	7 (39)	8 (30)	19 (30)		
Location if unilateral	Left	38 (51)	5 (46)	9 (47)	24 (55)	0.797	0.08 (0.04–0.36)
	Right	36 (49)	6 (55)	10 (53)	20 (46)		
Preoperative GCS	Mild	82 (87)	14 (100)	23 (92)	45 (82)	0.525	0.15 (0.10–0.26)
	Moderate	8 (9)	0 (0)	1 (4)	7 (13)		
	Severe	4 (4)	0 (0)	1 (4)	3 (6)		
CT density	Hypo	2 (2)	1 (8)	0 (0)	1 (2)	0.359	0.15 (0.07–0.39)
	ISO	8 (9)	2 (17)	2 (8)	4 (7)		
	Hyper	0 (0)	0 (0)	0 (0)	0 (0)		
	Mixed	81 (89)	9 (75)	23 (92)	49 (91)		
Preoperative maximum thickness	Mean (SD)	26.2 (12.6)	20.2 (12.9)	24.3 (16.2)	28.6 (10)	**0.016∗**	0.11 (0.00–0.24)
	Median (Q1–Q3)	25.5 (17.8–34)	10 (17.6–27)	15.5 (20–26.5)	29 (20–35)		
	Min–Max	2.5–85	2.5–43	10–85	6–56		
Median = 25.5	<25.5	35 (50)	7 (70)	13 (68)	15 (37)	**0.033**	0.32 (0.15–0.55)
	≥25.5	35 (50)	3 (30)	6 (32)	26 (63)		
Postoperative maximum thickness	Mean (SD)	14.5 (9.5)	11.5 (8.2)	10.6 (7.3)	17.1 (10.1)	**0.035∗**	0.09 (0.00–0.22)
	Median (Q1–Q3)	14 (7.8–21)	11.5 (6–17)	8 (5–14.5)	14 (10.5–25.5)		
	Min-Max	0–48	0–24	0–25	0–48		
Median SD = 14	<14	33 (47)	5 (50)	12 (63)	16 (39)	0.229	0.21 (0.05–0.46)
	≥14	37 (53)	5 (50)	7 (37)	25 (61)		
Preoperative midline shift	Mean (SD)	7.4 (5)	7.7 (5.9)	7.9 (5.3)	7 (4.8)	0.794∗∗	0.01 (0.00–0.07)
	Median (Q1–Q3)	7 (4–11)	6 (3.5–13)	7 (5–11)	7 (4–11.8)		
	Min-Max	0–18	0–15	0–18	0–18		
Median = 7	<7	33 (49)	4 (57)	10 (48)	19 (48)	0.938	0.06 (0.03–0.35)
	≥7	35 (52)	3 (43)	11 (52)	21 (53)		
Postoperative midline shift	Mean (SD)	2.6 (2.6)	2.6 (2.8)	2.6 (3.3)	2.7 (2.3)	0.851∗	0.01 (0.00–0.08)
	Median (Q1-Q3)	3 (0–4)	2 (0–5)	2 (0–4)	3 (0–4)		
	Min–Max	0–13	0–6	0–13	0–7		
Median = 3	<3	32 (47)	4 (57)	11 (52)	17 (43)	0.716	0.11 (0.03–0.37)
	≥3	36 (53)	3 (43)	10 (48)	23 (48)		
Clinical presentation:	Headache	70 (68)	14 (78)	22 (92)	34 (56)	**0.004**	0.33 (0.18–0.49)
	Limb weakness	42 (41)	3 (17)	6 (25)	33 (54)	**0.004**	0.33 (0.18–0.51)
	Speech impairment	7 (7)	0 (0)	0 (0)	7 (12)	0.136	0.22 (0.13–0.30)
	Unsteady gait	13 (13)	0 (0)	1 (4)	12 (20)	**0.033**	0.26 (0.15–0.38)
	Altered consciousness	21 (20)	1 (6)	2 (8)	18 (30)	**0.026**	0.27 (0.14–0.42)
Comorbidities:	HTN	59 (55)	0 (0)	13 (48)	46 (74)	**≤0.001**	0.55 (0.43–0.68)
	DM	36 (34)	0 (0)	8 (30)	28 (45)	**0.001**	0.35 (0.26–0.47)
	Heart diseases	39 (36)	1 (6)	9 (33)	29 (47)	**0.006**	0.31 (0.19–0.46)
Trauma		29 (27)	3 (17)	5 (19)	21 (33)	0.231	0.17 (0.04–0.35)
Smoking		21 (19)	2 (11)	6 (22)	13 (21)	0.703	0.10 (0.03–0.28)
Medications:	Anti-platelets	35 (33)	0 (0)	13 (48)	22 (36)	**0.003**	0.33 (0.25–0.47)
	Anti-coagulants	12 (11)	1 (6)	5 (19)	6 (10)	0.432	0.14 (0.03–0.37)
Recurrence		14 (13)	2 (11)	2 (7)	10 (16)	0.673	0.11 (0.03–0.28)

∗ p-values were computed using the Kruskal–Wallis test. Thus effect sizes are eta-squared (η^2^).

∗∗ p-values were computed using analysis of variance. Thus effect sizes are eta-squared (η^2^).

Values in bold highlight significant results (P value ≤ 0.05).

#### II. Unilateral versus bilateral

Of the patients with maximum thickness data, both the pre-operative and post-operative maximum thickness in the bilateral group (M = 32.9 and 20.8, SD = 15.3 and 10, respectively) were significantly higher than those of the unilateral group (M = 22 and 10.5, SD = 8.3 and 6.8) with medium and large effects, p = 0.001 (r = 0.41, 95% CI: 0.19–0.59) and ≤0.001 (r = 0.54, 95% CI: 0.35–0.69), respectively. In patients with midline shift data, the pre-operative midline shift in the unilateral group (M = 8.3, SD = 5.3) was higher than that of the bilateral one (M = 5.6, SD = 4) with statistical significance and a medium effect, p = 0.027 (d = 0.58, 95% CI: 0.07–1.08).

Regarding the clinical presentation, only limb weakness and nausea/vomiting proportions significantly differed, where limb weakness was more common in the unilateral group (48.6% vs. 24.2%; V = 0.23 “medium effect” [95% CI: 0.05–0.40], p = 0.019), while nausea/vomiting was more common in the bilateral group (51.5% vs. 20%; V = 0.32 “medium effect” [95% CI: 0.11–0.51], p = 0.001).

When comparing the comorbidities, none of them significantly differed. With respect to medication history, the bilateral group had a significantly higher proportion of patients with a history of anti-coagulants (22.9% vs 5.2%; V = 0.27 “medium effect” [95% CI: 0.05–0.46], p = 0.017).

Lastly, there was no statistical significance regarding the rate of recurrence between the two groups, (14.3% in the bilateral group vs. 11.2% in the unilateral one; V = 0.04 “small effect” [95% CI: 0.00–0.25], p = 0.762).

All of the results with their effect sizes for comparisons based on laterality are available in Table 3.

**Table 3:** All laterality-based comparisons effect sizes.

		Total	Unilateral	Bilateral	p-value	Effect sizes (95% CI)
			74	34		
Age	Mean (SD)	60.9 (22.1)	61.8 (21)	58.8 (24.5)	0.965∗∗	0.14 (−0.27–0.55)
	Median (Q1–Q3)	68.5 (48.3–76)	68 (50.5–76)	70.5 (46.3–76.3)		
	Min–Max	0.3–98	0.3–98	0.6–86		
	≤40	18 (17)	11 (15)	7 (21)	0.759	0.07 (0.03–0.30)
	41–64	27 (25)	19 (26)	8 (24)		
	≥65	63 (58)	44 (60)	19 (56)		
Sex	Male	76 (70)	51 (69)	25 (74)	0.626	0.05 (0.00–0.24)
Preoperative GCS	Mild	82 (87)	55 (89)	27 (84)	0.778	0.08 (0.03–0.31)
	Moderate	8 (9)	5 (8)	3 (9)		
	Severe	4 (4)	2 (3)	2 (6)		
CT density	Hypo	2 (2)	2 (3)	0 (0)	0.281	0.19 (0.08–0.31)
	Iso	8 (9)	7 (12)	1 (3)		
	Hyper	0 (0)	0 (0)	0 (0)		
	Mixed	81 (89)	50 (85)	31 (97)		
Preoperative maximum thickness	Mean (SD)	26.2 (12.6)	22 (8.3)	32.9 (15.3)	**0.001∗**	0.41 (0.19–0.59)
	Median (Q1–Q3)	25.5 (17.8–34)	23 (16–29)	34 (20.2–40)		
	Min–Max	2.5–85	2.5–36	10–85		
Median = 25.5	<25.5	35 (50)	27 (63)	8 (30)	**0.007**	0.32 (0.10–0.55)
	≥25.5	35 (50)	16 (37)	19 (70)		
Postoperative maximum thickness	Mean (SD)	14.5 (9.5)	10.5 (6.8)	20.8 (10)	**≤0.001∗**	0.54 (0.35–0.69)
	Median (Q1–Q3)	14 (7.8–21)	10 (5–14)	22 (15–26)		
	Min–Max	0–48	0–31	0–48		
Median = 14	<14	33 (47)	29 (67)	4 (15)	**0.001**	0.51 (0.33–0.69)
	≥14	37 (53)	14 (33)	23 (85)		
Preoperative midline shift	Mean (SD)	7.4 (5)	8.3 (5.3)	5.6 (4)	**0.027∗∗**	0.58 (0.07–1.08)
	Median (Q1–Q3)	7 (4–11)	9 (4.1–12)	5.5 (2.6–9.5)		
	Min–Max	0–18	0–18	0–13		
Median = 7	<7	33 (49)	17 (39)	16 (67)	**0.027**	0.27 (0.04–0.48)
	≥7	35 (52)	27 (61)	8 (33)		
Postoperative midline shift	Mean (SD)	2.6 (2.6)	2.8 (2.4)	2.3 (3.1)	0.243∗	0.14 (−0.10–0.37)
	Median (Q1–Q3)	3 (0–4)	3 (0–4.9)	2 (0–4)		
	Min-Max	0–13	0–7	0–13		
Median = 3	<3	32 (47)	18 (41)	14 (58)	0.169	0.17 (0.01–0.38)
	≥3	36 (53)	26 (59)	10 (42)		
Clinical presentation	Headache	70 (68)	46 (66)	24 (73)	0.477	0.07 (0.00–0.26)
	Limb weakness	42 (41)	34 (49)	8 (24)	**0.019**	0.23 (0.05–0.40)
	Nausea/vomiting	31 (30)	14 (20)	17 (52)	**0.001**	0.32 (0.13–0.51)
	Dizziness	11 (11)	9 (13)	2 (6)	0.496	0.10 (0.01–0.25)
	Speech impairment	7 (7)	6 (9)	1 (3)	0.425	0.10 (0.01–0.22)
	Unsteady gait	13 (13)	7 (10)	6 (18)	0.340	0.12 (0.01–0.33)
	Incontinence	8 (8)	6 (9)	2 (6)	1.00	0.04 (0.00–0.21)
	Altered consciousness	21 (20)	15 (21)	6 (18)	0.703	0.04 (0.00–0.23)
	Others	17 (17)	10 (14)	7 (21)	0.377	0.09 (0.00–0.29)
Comorbidities	HTN	59 (55)	44 (60)	15 (44)	0.118	0.15 (0.01–0.34)
	DM	36 (34)	28 (38)	8 (24)	0.131	0.15 (0.02–0.33)
	Heart diseases	39 (36)	25 (34)	14 (41)	0.488	0.07 (0.01–0.25)
	Cerebrovascular diseases	24 (22)	18 (25)	6 (18)	0.418	0.08 (0.00–0.26)
	Neuropathies (non-vascular)	15 (14)	7 (10)	8 (24)	0.073	0.19 (0.02–0.39)
	Nephropathies	5 (5)	3 (4)	2 (6)	0.652	0.04 (0.00–0.24)
	Cancers	5 (5)	2 (3)	3 (9)	0.324	0.13 (0.01–0.32)
	Metabolic disorders	10 (9)	7 (10)	3 (9)	1.00	0.01 (0.00–0.21)
	Others	13 (12)	8 (11)	5 (15)	0.751	0.05 (0.00–0.25)
Trauma		29 (27)	18 (24)	11 (32)	0.382	0.08 (0.00–0.26)
Smoking		21 (19)	12 (16)	9 (27)	0.211	0.12 (0.01–0.33)
Medications	Anti-platelets	35 (33)	22 (30)	13 (38)	0.406	0.08 (0.00–0.26)
	Anti-coagulants	12 (11)	4 (6)	8 (24)	**0.017**	0.27 (0.05–0.46)
	Preoperative steroids	3 (9)	3 (13)	0 (0)	0.550	0.12 (0.06–0.18)
Recurrence		14 (13)	9 (12)	5 (15)	0.762	0.04 (0.00–0.23)

∗ p-values were computed using the Mann–Whitney U test. Thus effect sizes are r statistics.

∗∗ p-values were computed using the Student's *t*-test. Thus effect sizes are Cohen's d.

Values in bold highlight significant results (P value ≤ 0.05).

#### III. Recurrence versus non-recurrence

The mean age of the recurrence group (M = 65.6, SD = 19.4) was higher than that of the non-recurrence one (M = 60.2, SD = 22.5), but with no statistical significance and a small effect, p = 0.356 (d = 0.26, 95% CI: −0.32–0.81). For patients with maximum thickness data, the means of the pre- and post-operative maximum thickness were both higher in the recurrence group (M = 28.6 and 15.7, SD = 8.7 and 10.8, respectively) compared to the non-recurrence one (M = 25.9 and 14.3, SD = 13.1 and 9.4), but with no significant difference and small effects in both, p = 0.287 (r = 0.13, 95% CI: −0.11–0.35) and 0.733 (r = 0.04, 95% CI: −0.20–0.27), respectively.

Regarding the midline shift, the mean pre-operative midline shift was significantly higher in the recurrence group (M = 11.3, SD = 4.4) compared with the non-recurrence one (M = 6.8, SD = 4.9) with a medium effect as well, p = 0.021 (r = 0.28, 95% CI: 0.05–0.49). Similarly, the mean post-operative midline shifting was also higher in the recurrence group (M = 3.8, SD = 1.8) compared to the non-recurrence one (M = 2.5, SD = 2.7), with no statistical significance, and a small effect, p = 0.067 (r = 0.22, 95% CI: −0.02–0.44). Lastly, comorbidities and medication history were not found to be associated with recurrence.

All of the results with their effect sizes for comparisons based on recurrence are available in Table 4.

**Table 4:** All recurrence-based comparisons effect sizes.

		Total	Recurrence	Non-recurrence	p-value	Effect sizes (95% CI)
			14	94		
Age	Mean (SD)	60.9 (22.1)	65.6 (19.4)	60.2 (22.5)	0.356∗∗	0.26 (−0.32–0.81)
	Median (Q1–Q3)	68.5 (48.3–76)	71 (53.3–80.5)	68 (47–76)		
	Min–Max	0.3–98	26–89	0.3–98		
	≤40	18 (17)	2 (14)	16 (17)	0.673	0.11 (0.03–0.28)
	41–64	27 (25)	2 (14)	25 (27)		
	≥65	63 (58)	10 (71)	53 (56)		
Sex	Male	76 (70)	11 (79)	65 (69)	0.549	0.07 (0.00–0.22)
CT density	Hypo	2 (2)	0 (0)	2 (3)	1.00	0.06 (0.02–0.24)
	Iso	8 (9)	1 (8)	7 (9)		
	Hyper	0 (0)	0 (0)	0 (0)		
	Mixed	81 (89)	11 (92)	70 (89)		
Preoperative maximum thickness	Mean (SD)	26.2 (12.6)	28.6 (8.7)	25.9 (13.1)	0.287∗	0.13 (−0.11–0.35)
	Median (Q1–Q3)	25.5 (17.8–34)	30 (22.6–35.5)	25 (17–33.5)		
	Min–Max	2.5–85	12–40	2.5–85		
Median = 25.5	<25.5	35 (50)	3 (33)	32 (53)	0.477	0.13 (0.01–0.34)
	≥25.5	35 (50)	6 (67)	29 (48)		
Postoperative maximum thickness	Mean (SD)	14.5 (9.5)	15.7 (10.8)	14.3 (9.4)	0.733∗	0.04 (−0.2–0.27)
	Median (Q1–Q3)	14 (7.8–21)	14 (7.5–27)	14 (7.5–19.3)		
	Min–Max	0–48	0–30	0–48		
Median = 14	<14	33 (47)	4 (44)	29 (48)	1.00	0.02 (0.00–0.26)
	≥14	37 (53)	5 (56)	32 (53)		
Preoperative midline shift	Mean (SD)	7.4 (5)	11.3 (4.4)	6.8 (4.9)	**0.021∗**	0.28 (0.05–0.49)
	Median (Q1–Q3)	7 (4–11)	11.5 (8.3–14.2)	6 (3.3–10.8)		
	Min-Max	0–18	4–18	0–18		
Median = 7	<7	33 (49)	1 (13)	32 (53)	0.055	0.26 (0.06–0.44)
	≥7	35 (52)	7 (88)	28 (47)		
Postoperative midline shift	Mean (SD)	2.6 (2.6)	3.8 (1.8)	2.5 (2.7)	0.067∗	0.22 (−0.02–0.44)
	Median (Q1–Q3)	3 (0–4)	4 (3.3–4.8)	2.3 (0–4)		
	Min–Max	0–13	0–6	0–13		
Median = 3	<3	32 (47)	1 (13)	31 (52)	0.058	0.25 (0.06–0.41)
	≥3	36 (53)	7 (88)	29 (48)		
Comorbidities:	HTN	59 (55)	9 (64)	50 (54)	0.461	0.07 (0.00–0.25)
	DM	36 (34)	6 (43)	30 (32)	0.546	0.08 (0.00–0.28)
	Heart diseases	39 (36)	7 (50)	32 (34)	0.258	0.11 (0.01–0.31)
Medications:	Anti-platelets	35 (33)	5 (36)	30 (32)	0.769	0.03 (0.00–0.22)
	Anti-coagulants	12 (11)	2 (14)	10 (11)	0.656	0.04 (0.00–0.28)
	Preoperative steroids	3 (9)	1 (17)	2 (7)	0.346	0.10 (0.03–0.39)
	Postoperative steroids	8 (22)	1 (17)	7 (23)	1.00	0.01 (0.00–0.22)

∗ p-values were computed using the Mann–Whitney U test. Thus effect sizes are r statistics.

∗∗ p-values were computed using the Student's *t*-test. Thus effect sizes are Cohen's d.

Values in bold highlight significant results (P value ≤ 0.05).

## Discussion

CSDH is one of the most encountered intracranial hemorrhages in the neurosurgical practice, especially in the elderly population.^1^ However, we did not find any previous study that represents the Middle Eastern population where the mean age is lower than that in developed countries.^30^ In this study, we described the clinical presentation, radiological findings, surgical management, and post-operative outcomes in KAUH, northern Jordan.

The reported mortality rate ranges from 0.2% to 32% and the complication rate is 0–38%.^7^^,^^26^^,^^31^ In our study, the mortality and complication rates were 2.8% and 9.3%, respectively. The mean age in our study was 60.9 years, which is younger than that reported in Western developed countries but consistent with other developing countries.^23^^,^^32^^,^^33^ This finding may be due to a younger average age of the general Jordanian population.^30^ As expected, most of the patients were men with a male to female ratio of 2.38:1; 27.1% of patients had a history of trauma, which was lower than that previously reported.^10^^,^^12^^,^^13^^,^^32^ Most of the trauma cases were in the ≥65 age group (72.4%). The percentage of HTN and DM was 55.1% and 33.7%, respectively; this was higher than other studies, which might be explained by the higher prevalence of both diseases in Jordan.^34^^,^^35^ This might explain why trauma is less likely to be an etiological factor for CSDH and suggests hypertension as a possible risk factor for CSDH in our study, especially because only 13.56% were adherent to their anti-hypertension medications.^36^

Headache was the most common presenting symptom in our cohort, which was similar to other studies.^32^^,^^37^ However, it was more significant in patients < 65 years. Limb weakness, unsteady gait, and altered consciousness were significant in patients ≥ 65 years. This may indicate the severity of the disease in patients ≥ 65 years on presentation to our hospital.

The incidence of bilateral CSDH was 31.5%, similar to the reported incidences of 9.2–34.9%.^26^^,^^38^ However, recurrence was not as significant as in other studies,^17^^,^^18^ possibly due to the small sample size of our study. Patients with bilateral CSDH more commonly presented with nausea than patients with unilateral CSDH. Interestingly, 81 patients had mixed density appearance on CT scan with no patients having a hyperdense appearance. These results differ from what has been reported.^14^^,^^26^

BHC is the surgical treatment most frequently employed for CSDH.^39^ Most of our patients underwent closed system BHD, whereas the rest underwent craniotomy. In a systematic review and meta-analysis done by Lega et al.,^40^ BHD and craniotomy were similar in terms of complications, but recurrence rate was lower in the craniotomy group. The authors concluded BHD is the most efficient choice for managing CSDH taking into account both recurrence rate and complications. However, in another systematic review and meta-analysis performed by Ducruet et al.,^22^ the authors recommended the use of twist drill craniostomy for high-risk surgical patients with unseptated CSDH, while craniotomy in the presence significant membranes was associated with a lower risk of recurrence and post-operative mortality but with a higher risk of complications compared with craniotomy. The use of twist-drill drainage as a first-line option was also supported by Almenawer et al.^9^ The optimum modality is still debatable and further studies are needed.

We found that recurrence was not associated with age, bilaterality, CT density, pre- and post-operative maximum thickness, comorbidities, clinical presentation, perioperative steroids use, medications, and post-operative midline shift. Ridwan et al.^13^ demonstrated similar results except for anti-platelet therapy. Only pre-operative mean midline shift was a significant indicator of recurrence in our study (p = 0.021). By contrast, many studies have reported a risk of recurrence associated with factors such as age, GCS, HTN, and DM.^8^^,^^10^, ^11^, ^12^

Our study had some limitations including the retrospective design of the study; the small number of patients, which rendered us unable to perform multivariate analyses due to the possibility of low precision; the loss of patients during the follow-up period; and the hospital's limited geographical coverage.

## Conclusion

In summary, we described the clinical presentation, surgical management, and post-operative outcomes in KAUH. The main surgical modality used was a closed system BHD with low mortality and post-operative complications. Patients < 65 years commonly presented with headaches, while patients ≥ 65 years presented with limb weakness, speech impairment, unsteady gait, and altered consciousness. Only pre-operative mean midline shift was significantly associated with recurrence. Further large-scale studies are needed to more clearly define the classic CSDH patient and the risk factors associated with recurrence in a Middle Eastern population.

## Source of funding

This research did not receive any grant from funding agencies in the public, commercial, or not-for-profit sectors.

## Conflict of interest

The authors have no conflicts of interest to declare.

## Ethical approval

This work was approved by the institutional review board of King Abdullah University Hospital (Ref. No. 102/132/2020, date 21.04.2020).

## Authors contributions

SJ Conceptualization, methodology, supervision, project administration, writing – review and editing. MB Conceptualization, resources, supervision. SD Conceptualization, resources. QS Methodology, resources, writing – original draft, writing – review and editing. AQ Investigation, data curation, writing – original draft, writing – review and editing. RA Data Curation, writing – original draft, writing – review and editing. NA Formal analysis, writing – original draft, writing – review and editing. OA Investigation, data curation, writing – original draft, writing – review and editing. OJ supervision, investigation, methodology, resources, writing – original draft, writing – review and editing. All authors have critically reviewed and approved the final draft and are responsible for the content and similarity index of the manuscript.
